# Tumor-initiating and metastasis-initiating cells of clear-cell renal cell carcinoma

**DOI:** 10.1186/s12929-024-01111-9

**Published:** 2025-02-08

**Authors:** Dinh-Xuan Pham, Tien Hsu

**Affiliations:** 1 Department of Biomedical Sciences and Engineering, National Central University, Taoyuan, Taiwan, ROC; 2 Graduate Institute of Biomedical Sciences, China Medical University-Taiwan, No. 91 Hsueh-Shih Road, Taichung, 40402 Taiwan, ROC

**Keywords:** Clear-cell renal cell carcinoma, Cancer stem cells, Tumor-initiating cells, Metastasis-initiating cells, Kidney progenitor cells, *VHL* tumor suppressor gene

## Abstract

Clear-cell renal cell carcinoma (ccRCC) is the most common subtype of kidney malignancy. ccRCC is considered a major health concern worldwide because its numbers of incidences and deaths continue to rise and are predicted to continue rising in the foreseeable future. Therefore new strategy for early diagnosis and therapeutics for this disease is urgently needed. The discovery of cancer stem cells (CSCs) offers hope for early cancer detection and treatment. However, there has been no definitive identification of these cancer progenitors for ccRCC. A majority of ccRCC is characterized by the loss of the *von Hippel-Lindau* (*VHL*) tumor suppressor gene function. Recent advances in genome analyses of ccRCC indicate that in ccRCC, tumor-initiating cells (TICs) and metastasis-initiating cells (MICs) are two distinct groups of progenitors. MICs result from various genetic changes during subclonal evolution, while TICs reside in the stem of the ccRCC phylogenetic tree of clonal development. TICs likely originate from kidney tubule progenitor cells bearing *VHL* gene inactivation, including chromatin 3p loss. Recent studies also point to the importance of microenvironment reconstituted by the *VHL*-deficient kidney tubule cells in promoting ccRCC initiation and progression. These understandings should help define the progenitors of ccRCC and facilitate early detection and treatment of this disease.

## Background

Clear-cell renal cell carcinoma (ccRCC) constitutes the majority (up to 70%) of primary RCC [1–3]. Many ccRCC patients present notable symptoms (hematuria, anemia, cachexia, and flank pain) only in advanced stages [4], making early treatment difficult. The majority (50–60%) of ccRCC cases are diagnosed incidentally via noninvasive imaging, and 30–50% of the cases are diagnosed at metastatic stages [5]. Notably, while the 5-year survival rate of early-stage ccRCC can be up to 90%, that of metastasized ccRCC is only about 12% [5]. These statistics point to the need for early detection and treatment of ccRCC.

The first critical genetic event of sporadic ccRCC is the haploid loss of the short arm of chromosome 3 (3p loss), which is detected in almost 90% of patients [6, 7]. The genomic region of 3p loss encompasses four well-recognized tumor suppressor genes (*VHL*, *PBRM1*, *BAP1*, and *SETD2*) [8–11]. Inactivating mutations (loss-of-heterozygosity) or epigenetic changes (mainly promoter hypermethylation) of the tumor suppressor gene *VHL* in particular are the main drivers of ccRCC, while loss of *PBRM1, BAP1* or *SETD2* is subordinate to *VHL* loss [12, 13]. Interestingly, *PBRM1, BAP1,* and *SETD2* are involved in chromatin remodeling, suggesting that widespread epigenetic changes, not specific genetic mutations besides those in *VHL*, can contribute to ccRCC formation.

Currently, computed tomography and magnetic resonance imaging are the mainstays of ccRCC diagnosis [14, 15]. Yet, these two methods’ clinical application and prediction process are costly and still largely dependent on subjective human interpretation. One obstacle to accessible diagnostic strategy is that there are as yet no proven biomarkers for early-stage ccRCC. Even though various potential markers have been proposed, very few proved useful in clinical settings [16–18]. One promising diagnostic strategy may be based on the discovery that early ccRCC shared common serum/urinary inflammatory signatures with chronic kidney disease (CKD). Indeed, mounting evidence has implicated tissue inflammation in the tumorigenesis of ccRCC [19–23], and CKD has proved an important risk factor for ccRCC [24, 25]. However, accessible methods that can differentiate inflammatory kidney disease and early kidney cancer remain elusive. For this purpose, the presence of cancer stem/progenitor cells, combined with kidney inflammatory markers may offer an opportunity for early diagnosis [26–28]. The potential inflammatory markers include interleukin-6 (IL-6), a prominent tissue and serum inflammatory cytokine [29, 30]; kidney injury molecule-1 [KIM-1, also known as T-cell Ig and mucin domain-1 (TIM-1)], a serum and urine biomarker for human renal tubule injuries and kidney cancer [31, 32]; neutrophil gelatinase-associated lipocalin [NGAL, also known as lipocalin2 (LCN2)], a tissue and serum marker associated with inflammatory disease and cancer [33, 34]; and fibroblast growth factor 23 (FGF23), a growth factor involved in decreasing reabsorption of phosphate in the kidney and a marker for kidney disease [35, 36].

### Cancer stem cells: are they tumor-initiating or metastasis-initiating cells?

The CSC theory originated from the study of teratocarcinoma, in which the cancerous growth contains a mixture of differently differentiated cell types [37]. The theory suggests that there exists a self-renewing primordial cell population that gives rise to the tumor mass containing progenies with different degrees of differentiation, while the progenitor clone can also directly give rise to malignant cancer, hence the term CSC. The theory therefore can also explain the conundrum that in clinical settings, occasionally metastasis can occur before the primary tumor is detected.

Indeed, CSCs have now been identified in a wide range of cancers [38]. However, whether the currently used term CSCs truly indicates the progenitor cells that initiate the growth of a tumor remains unresolved. The debate is also still ongoing as to whether malignant cancer originates from CSCs or is the result of clonal evolution [39], since with the exception of rare fast growing, highly aggressive cancer cases, the development of cancer malignancy is time-dependent and can be correlated with the size of primary tumors. This suggests that the hierarchical clonal evolution model, as opposed to the model of preexisting CSCs, may still be valid. One of the problems likely lies in the interchangeable usage of CSCs to depict TICs and MICs, because of the unspecified distinction between the two populations. In the case of ccRCC, it has been observed that not all *VHL*-deficient cells develop into metastatic ccRCC [40], and loss of chromosomes 9p and 14q contributes to ccRCC metastasis subsequent to *VHL* loss [41]. This indicates that *VHL* loss is necessary for tumor growth but insufficient for metastasis. Therefore such distinction, as will be discussed in this review, is relevant in regard to ccRCC. TICs, or sometimes called cancer cells of origin, are tumorigenic cells exhibiting features of stem cells, whereas MICs, although born from TICs, foster additional attributes such as the spread and recurrence of malignancy [42].

In this review, we will use the term CSC only when the cited literature made no distinction between the origin of primary tumor and the origin of metastatic subclone. When appropriate, we will use TIC and MIC to specify the two events.

### The origin of cancer stem cells

**Fig. 1:**
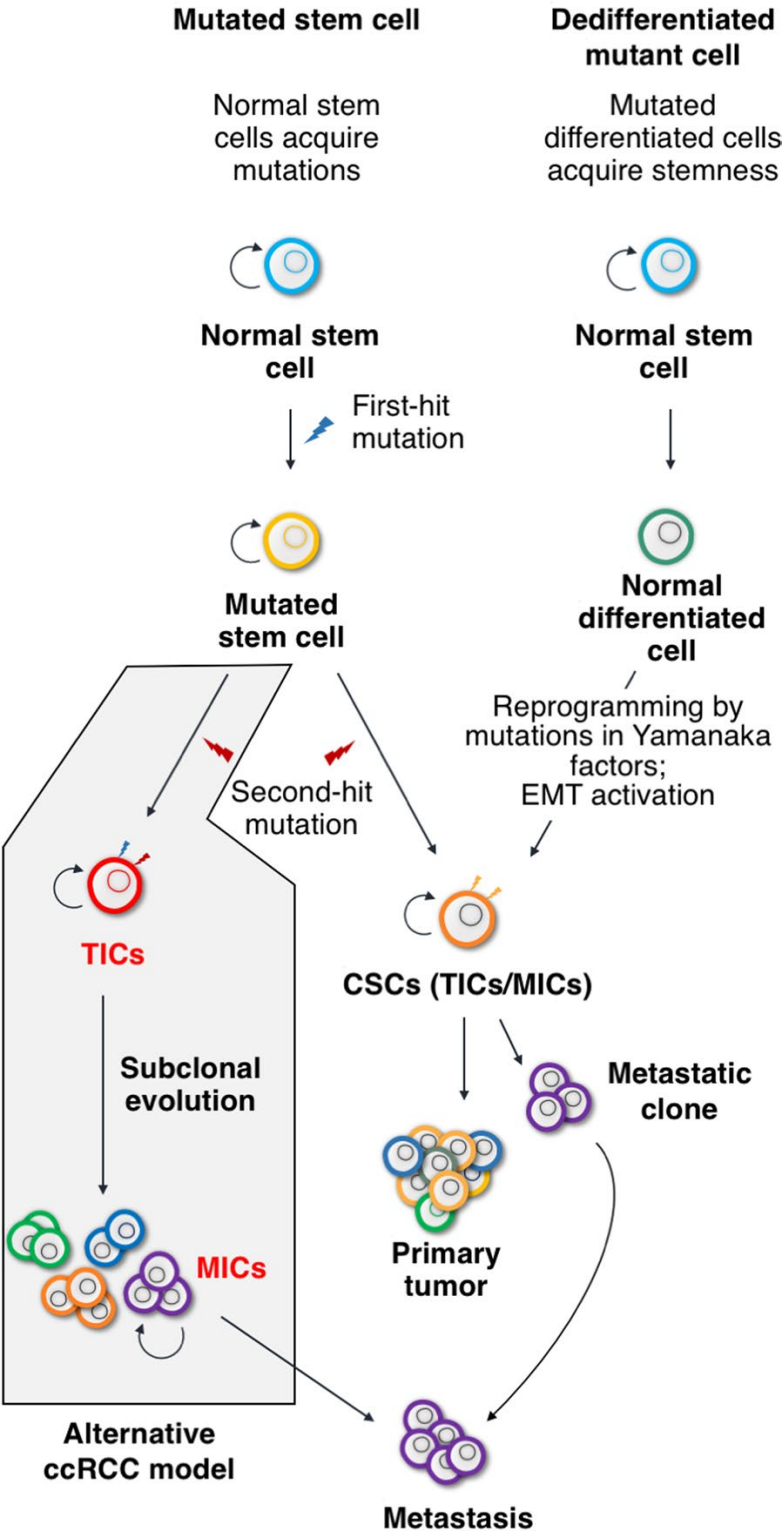
An alternative model of the origin of ccRCC. Two mechanisms are proposed to explain the origin of CSCs: either they are mutated stem cells (left) or dedifferentiated mutant cells (right). The former suggests that CSCs originate from adult stem cells that accumulate mutations. In the latter theory, cellular changes and microenvironmental factors can transform differentiated cells into malignant, dedifferentiated cells. The development of ccRCC is likely a hybrid model (shaded pathway on the left), in which normal stem cells with 2 hits in the *VHL* gene, one of which involves chromosome 3p loss, become TICs. The TICs then undergo subclonal evolution to generate metastatic subclone, which constitutes MICs

Two mechanisms have been proposed to account for the origin of CSCs: either they are mutated adult stem cells (normal stem cells that acquire mutations) or mutated differentiated cells that acquire progenitor features (Fig. 1). The former can be called “mutated stem cell” theory and the latter “dedifferentiated mutant cell” theory.

In the “mutated stem cell” theory, the origin of CSCs is adult stem cells that accumulate pro-tumorigenic mutations. From whole-genome sequencing of adult stem cells (clonal organoid cultures derived from primary multipotent cells) of the small intestine, colon, and liver of human donors with ages ranging from 3 to 87 years, it was revealed that mutations accumulate steadily over time, at a rate of approximately 40 mutations per year [43]. It is therefore conceivable that a “right hit,” or a combination of critical hits, in the adult tissue stem cells can render these stem cells tumorigenic. For example, deletion of *Apc* in long-lived Lgr5^+^ intestinal stem cells leads to transformation of the stem cells within days. The transformed stem cells remain at the crypt bottom, forming microadenomas exhibiting unimpeded growth, and become macroscopic adenomas within 3–5 weeks. Importantly, the same *Apc* deletion fails to drive intestinal adenoma formation when introduced in more differentiated cells [44].

In the “dedifferentiated mutant cell” theory, mutations accumulated in differentiated cells can induce cellular changes such as epithelial-to-mesenchymal transition (EMT) that transforms the benign cells into malignant, dedifferentiated cells. In a landmark study, Mani et al. [45] demonstrated that the transformed human mammary epithelial cells showed transplantable tumor formation and metastasis-initiating ability through activation of EMT (ectopic induction of TGF-β signaling or ectopic expression of either Twist or Snail transcription factors). This study reconciled two seemingly contradictory aspects of cancer initiation: if cancer stem cells exist at the beginning of cancer formation, why the development of malignant cancer is largely time-dependent? The answer therefore lies in the need to accumulate the “right” mutations that induce, in the case of Mani et al. study, EMT, or other oncogenic processes. Besides EMT, differentiated cells can also be reprogrammed to exhibit tumorigenic potential by activation of c-MYC and other “Yamanaka factors” including OCT3/4, SOX2, and KLF4 [46–50]. These findings also explain why in the studies using clonal selection of metastatic cancer cells, multiple and often inconsistent candidates of CSCs have been the result, since there may be more than one genetic pathways that can induce malignancy.

**Fig. 2:**
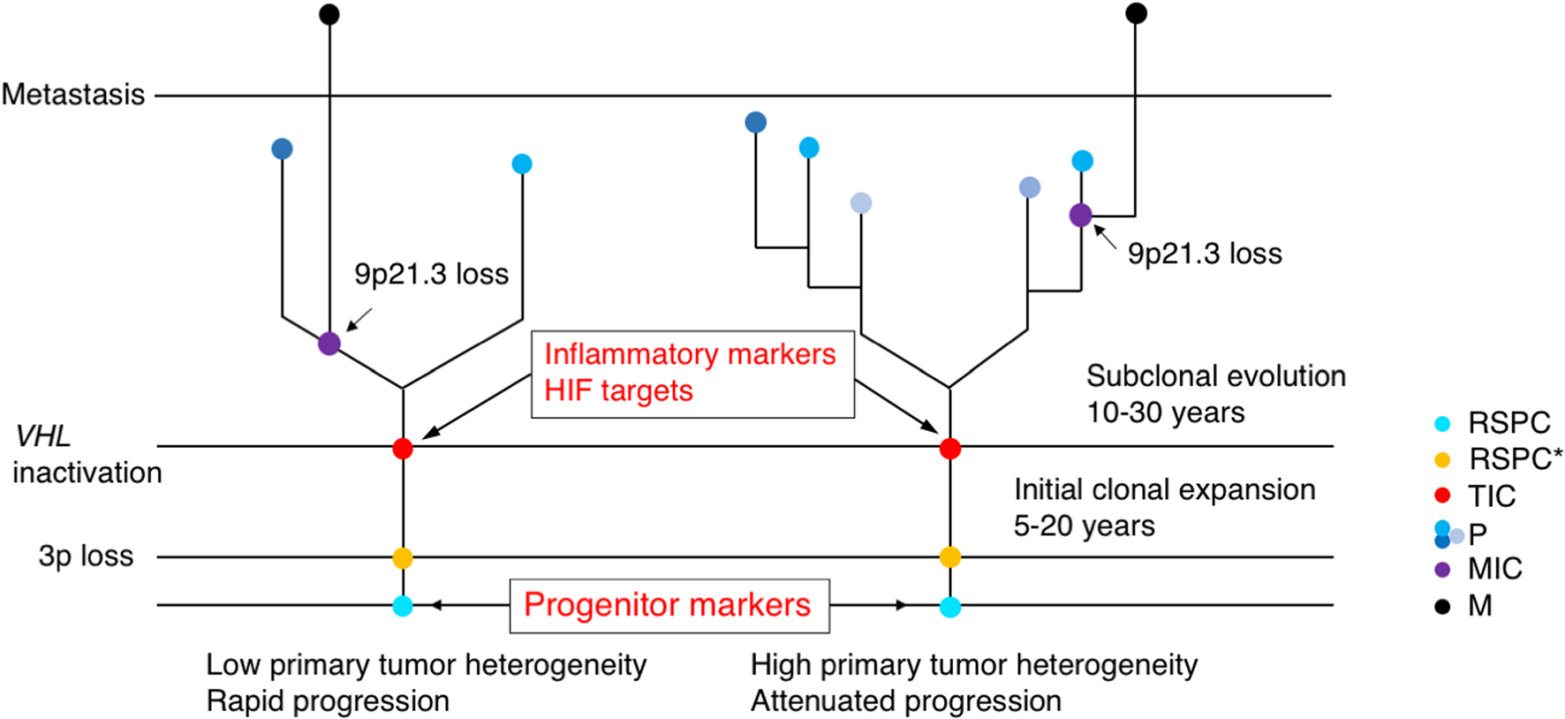
Genomic landscape of ccRCC tumor initiation and metastatic subclonal evolution. Two broadly defined scenarios can account for the initiation and progression of ccRCC. The RSPCs, expressing stem/progenitor cell markers such as Notch or Wnt signaling and CD133 or CD105, first acquire 3p loss (encompassing the *VHL* tumor suppressor gene), and begin a slow clonal expansion lasting 5–20 years before the appearance of TICs when the second allele of *VHL* is inactivated, which leads to expression of inflammatory markers such as KIM-1 and HIF targets such as CXCR4. The appearance of TICs initiates subclonal evolution that can last 10–30 years, giving rise to various genetically distinct benign subclones, before the emergence of MICs, which is often accompanied by the 9p21.3 loss. In rare cases, TICs, and hence MICs, can arise from *VHL*^+^ cells. These are not included in this general description. Early appearance (i.e., close to or on the phylogenetic stem) of the metastatic subclone characterizes low primary tumor heterogeneity and rapid progression of the disease (left), and late appearance (i.e., after multiple subclonal branching events) of metastatic subclone characterizes high primary tumor heterogeneity and slow progression of the disease (right). RSPC: renal stem/progenitor cell; RSTC*: pre-tumorigenic RSTC; TIC: tumor-initiating cell; P: heterogeneous primary tumor subclones; MIC: metastasis-initiating cell; and M: metastatic subclone. Adapted from Turajlic et al. [41] and Mitchell et al. [51]

The implication of these studies suggests that TICs and MICs reside in temporally different loci during progression of cancer. The genomic sequencing of a large cohort of ccRCC samples [41, 51], including normal-metastasis pairs, has suggested that an alternative model is likely the case for ccRCC (Fig. 1); that is, mutated stem cells give rise to TICs that form the stem of the phylogenetic tree of primary tumor growth, while dedifferentiated mutant cells initiate the process of subclonal evolution from which the metastatic subclone eventually emerges (Fig. 2). Therefore, the presence of TICs or their molecular signature can serve as early diagnostic markers and treatment targets; while MICs may be the targets for treatment against metastasis of already developed tumor mass. Next, we will discuss the nature of ccRCC stem/progenitor cells.

### CSCs of ccRCC—the current status

A number of studies have attempted to identify the CSCs of ccRCC, with varied and sometimes contradictory results. These have been reviewed previously [52–54] and are summarized in Table 1. One strategy is to use known stem cell/CSC markers to isolate RCC stem/progenitor cells from clinical samples or established cell lines using fluorescence-activated cell sorting (FACS) or magnetic-activated cell sorting (MACS). The markers often used include the following:

**Table 1:** Prior studies on CSCs of ccRCC

Studies	Approaches	Specimens^a^	Putative population	% Total tumor mass	Positive expression	Negative expression	CSC features	Reference
1	MACS	Human RCC nephrectomy sample (no clinical data provided)	CD105^+^	8.06 ± 3.3%	Nestin, NANOG, Musashi, OCT4, PAX2, CD29, CD44, CD73, CD90, CD146, Vimentin	Pan-CK, CD24, CD133	Clonogenic and sphere-forming ability; epithelial and endothelial differentiation in vitro; high tumorigenicity	Bussolati et. al. [66]
2	FACS	Human metastatic ccRCC biopsies (all VHL^−^) and patient-derived xenografts both VHL^−^ and VHL^+^)	CD133^+^/ CXCR4^+^	84.5% ± 2.5% in peri-necrotic area			Sphere formation; tumorigenicity; chemotherapy resistance (sunitinib)	• Varna et al. [69]
3	FACS	Human ccRCC biopsies (selected surface marker analysis of 55 patient samples, mainly pT1 and pT3; male:female = 1.8:1)	CXCR4^+^/ MET^+^/ CD44^+^	2.2%	VCAM1, E-Cadherin, KLF4, SOX9, PAX2, SALL1, PROM1 (CD133), ALDH1A1, MYST3		NOTCH and WNT signaling-dependent sphere- and organoid-formation; tumorigenicity	Fendler et al. [71]
4	MACS/ FACS	Normal and malignant biopsies from 40 ccRCC patients; pT1-pT3, male:female = 2.6:1	CD133^+^/ CD24^+^	85%	CTR2, NANOG, SOX2, CD73, SNAI1, VEGFR2, GATA-4, CD73	CD90, CD105, CD20, CD45, CD14, CD34	Self-renewal; multipotency in vitro*;* colony-formation in soft agar; angiogenic induction; CD133^+^/ CD24^+^ tumor cells less differentiated than normal progenitors	• Galleggiante et al. [67]
5	FACS	Caki-1	CD105^+^; CD44^+^; CD44^−^; CD44^−^/CD105^+^; CD105^−^/CD44^−^	10.8% CD105^+^, 1.68% CD133^+^, 94.6% CD44^+^	Multiple clones expressing combinations of positive or negative CD105 and CD44 markers		Tumorigenic potency (CD105^−^/CD44^−^ cells, not CD105^+^/ CD44^+^ or single CD105^−^cells); vasculogenic promotion	Fiedorowicz et al. [72]
6	MACS	ACHN, Caki-1	CD133^+^/ CD24^+^	ACHN, 95.8%; Caki-1, 95.5%	CTR2, BCL-2, MDR1, OCT-4, KLF4, NOTCH1, NOTCH2, JAG1, JAG2, DLL1, DLL4		NOTCH signaling-dependent self-renewal; chemotherapy resistance (cisplatin, sorafenib); tumorigenesis in vivo	Xiao et al. [70]
7	FACS	RCC-26, RCC-53	CXCR4^+^	RCC-26, 0.8%; RCC-53, 5%	OCT3/4, SOX2, NANOG, CD24, CD29, CD44, CD73, CD117, CD146, CXCR4	CXCR1, CD34, CD90, CD105, CD133	High ALDH1 activity; sphere-forming; tumorigenicity; RCC-53 cells, 100% incidence; resistance to RTK inhibitors	• Gassenmaier et al. [68]
8	Side population	Matched normal and malignant biopsies from ccRCC patients	Hoechst^−^	Normal kidney, 3.8 ± 0.4; RCC, 5.9 ± 0.9%	β-catenin, NOTCH1, SHH, CD133, PAX2		Highly proliferative; retention of SP upon culture; sphere formation in 3D Matrigel	Addla et al. [81]
9	Sphere formation assay, side population	SK-RC-42		9.9 ± 0.14% (SP)	OCT3/4, BMI1, β-catenin, NANOG	MHC-II, CD80, CD86	Sphere-formation; chemo-and radiotherapy-resistance (5-FU, MMC and 2 Gy); tumorigenicity	Zhong et al. [85]
10	Side population	769-P, 786-O, OS-RC-2, SN12C, SKRC39	Hoechst^−^	769-P, 4.82% (SP)	ABCB1	ABCG2, ABCC1	Self-renewal and proliferative potential; chemo-and radiotherapy-resistance (5-FU, MTX, 5 Gy); high tumorigenicity	Huang et al. [86]
11	Side population	ACHN, Caki1, SMKTR2, SMKTR3, RenCa	Hoechst^−^	ACHN, 2.6%; RenCa, 18% (SP)	DNAJB8 Sox2 Oct4 (Pou5f1)		Tumorigenicity	Nishizawa et al. [87]
12	Side population	ACHN, KRC/Y		ACHN, 1.4%; KRC/Y, 1.7%	ALDH1	CD105, CD133	Sphere formation, self-renewal, tumorigenicity (in ACHN but not KRC/Y)	Ueda et al. [88]
13	Sphere formation assay	ACHN, Caki-1			OCT4, NANOG, LIN28, KLF4, CD24, CD44, ZEB1, ZEB2, TWIST1, N-cadherin, Vimentin		TGF-β-dependent sphere formation; self-renewal; tumorigenicity	Lichner et al. [90]
14	Sphere formation	Caki-1, 786-O, (Caki-2, 769-P)			CXCR4, SDF-1, NANOG		Sphere formation; adipogenic differentiation; tumorigenicity (Caki-2, 769-P had none of the above)	Micucci et al. [91]

^a^For pathological and genetic features of the cell lines, refer to Wolf et al. [92], Brodaczewska et al. [93], and Sanger Institute COSMIC database; 5-FU, 5-fluorourocil, cytotoxic agent; cisplatin, DNA-chelating genotoxic agent; FACS: fluorescence-activated cell sorting; MACS: magnetic-activated cell sorting; MMC: mitomycin C; MTX: methotrexate; MMC, mitomycin-C, genotoxin, promotes DNA crosslinking; MTX, methotrexate, inhibitor of thymidine synthesis; RTK: receptor tyrosine kinase; sorafenib, RTK inhibitor; sunitinib; RTK inhibitor

CD105, also named Endoglin, is a receptor for TGF-β and therefore is presumed to promote EMT in stem cell formation. It is a recognized stem cell marker because of its identification as highly expressed in mesenchymal stem cells (MSCs) [55]. It is subsequently found to be overexpressed in multiple malignant cancers. However, the usefulness of relying on CD105 for identifying CSCs or TICs may be questioned since CD105^−^ MSCs also exist [56] (also see below).

CD133, also named Prominin-1, is a surface marker of hematopoietic stem cells and endothelial cells, and subsequently found to be expressed in multiple CSCs [57]. It can promote self-renewal by activating MAPK, PI3K/AKT, and WNT signaling pathways. It is also highly expressed in metastatic cancer cells. However, not all CSCs express CD133 [58].

CD44 is a receptor for hyaluronan and osteopontin, and is overexpressed in metastatic and stem cells [59]. It is known to promote EMT and can anchor stem cells in the niche. As a CSC marker, it is often combined with the expression of CD24 [60, 61]. However, there seems to be low predictability for cancer stemness since either high or low expression of CD44/CD24 combination can be found in CSCs in different contexts [62, 63].

CD24 is a P-selectin receptor and is a marker found in multiple CSCs. As stated above, it is often used for CSC identification in combination with CD44, although its accuracy in ccRCC progenitor identification is yet to be fully assessed [62, 63].

CXCR4 is a hypoxia- and hypoxia-inducible factor (HIF)-induced receptor for the chemokine CXCL12/stromal cell-derived factor-1 (SDF-1), and is therefore a good marker for *VHL* mutant tumor cells. It has been implicated in stem cell retention in the stem cell niche as well as in stem cell mobilization, depending on the source of the ligand CXCL12/SDF-1—whether the ligand is expressed by the abutting niche cells or from the target tissue, respectively [64, 65].

These stem cell markers have been used to isolate CSCs of ccRCC (Studies 1–7 in Table 1) [66–72]. Particularly, CD133 has been suggested as a selective marker for resident progenitor cells in normal adult human kidney [73–75], which is an attractive attribute for CSCs. Although the tumorigenic role of CD133^+^ cells has been postulated in many solid malignancies, the precise function of these progenitor cells in renal carcinogenesis still remains unresolved [76–80]. The CD133-expressing cells were indeed enriched in the side population (SP) in both normal human kidney tissue and human RCC (Study 8 in Table 1) [81]. SP is considered stem cell-like because exclusion of DNA dye is a recognized phenotype of stem cells owing to their elevated efflux activity of ATP-binding cassette (ABC) transporter protein family that excretes dyes absorbed from the culture medium [82, 83]. These cells can be sorted as “side population” because they appear as a small group of cells low in DNA dye staining in FACS analysis. However, in co-transplantation with RCC cells, the tumor-derived CD133^+^ cells favored vascularization and enhanced tumor growth rather than initiating tumor growth of their own lineage in non-obese diabetic/severe combined immunodeficiency (NOD/SCID) mice [84]. It is probable that CD133^+^ cells represent a subset of renal progenitor cells or MSCs within the tumor, but not a TIC or MIC population. This is consistent with another study by the same research group, in which the highly tumorigenic human RCC-derived CD105-positive cells lack expression of CD133 (Study 1 in Table 1) [66]. Moreover, the expression of MSC and embryonic renal cell markers in these CD105^+^ clones suggests that the renal CSCs may not be of CD133^+^ origin, but rather originate from an undifferentiated CD105^+^ cell population that retains the MSC phenotype in the adult kidney. However, it is equally possible that CD105^+^ and CD133^+^ populations are MIC markers in different metastasis subclones and may be mutually exclusive.

Yet another study using RCC cell line SK-RC-42, which is derived from bone metastasis of unknown *VHL* status, showed a contradictory result in which CD105 was expressed in almost all monolayer adherent cells but was reduced in sphere-forming cells (Study 9 in Table 1) [85]. Conversely, a subpopulation of Caki-1 RCC cell line of *VHL* wild-type genotype and notably lacking both CD105 and CD44, displayed high tumorigenic potential when implanted into NOD/SCID mice (Study 5 in Table 1) [72].

Using functional assays such as SP detection and sphere formation may offer less biased criteria for isolating TICs, as opposed to utilizing preconceived stem cell markers. Some SP studies have indeed identified other potential stem cell markers. For example, in the SPs of various RCC cell lines (Study 10 in Table 1), ABCB1 transporter has been identified as a CSC marker of RCC [86], not surprising since the functional assay was based on the ABC activity. The human RCC cell lines ACHN, Caki-1, SMKTR2, SMKTR3, and murine RenCa cells were analyzed for expression of heat shock protein (HSP) 40 family member DnaJ (Hsp40) homolog, subfamily B, member 8 (DNAJB8) in SP (Study 11 in Table 1) [87]. Overexpression of DNAJB8 enhances the expression of stem cell markers and tumorigenicity. RT-PCR analysis of these isolated SP cells showed that DNAJB8 was predominantly coexpressed with Yamanaka factors such as SOX2 and OCT4/POU5F1. Western blotting and immunostaining using SP cells also corresponded with preferential expression of DNAJB8 protein, confirming the stem cell-like phenotypes [87]*.* In addition, SP from the cell line ACHN has identified Aldehyde dehydrogenase 1 gene (*ALDH1*) as a potential RCC stem cell marker (Study 12 in Table 1) [88], which has been implicated as a CSC marker for various other cancers possibly by providing increased drug resistance [89]. However, these SP cells from ACHN did not express CD105 or CD133, and the other cell line used in the same study, KRC/Y, although forming SP, did not show increased sphere-forming capacity or increased *ALDH1* expression. It is notable that both ACHN and KRC/Y cells are *VHL* wildtype, but KRY/C is not histologically ccRCC and overexpresses mutant *TP53*.

Other studies using functional assays such as sphere formation has also identified a number of markers in the putative CSCs in RCC cell lines, including EMT markers CXCR4, SDF-1, ZEBs, TWIST, N-cadherin, and Vimentin, as well as canonical stem cell markers such as OCT4, NANOG, KLF4, CD24, and CD44 (Studies 13 and 14 in Table 1) [90, 91].

Nonetheless, even in these functional assays results could be inconsistent. In one study, SP analysis (exclusion of Hoechst 33342) on different ccRCC cell lines yielded appreciable SP in only one (769-P) of 5 lines (Study 10 in Table 1) [86]. While in this study, ABCB1^+^ SP was identified in the 769-P cell line, no stem-like cells were isolated from the same cell line in another study using sphere formation as the identification criterion (Study 14 in Table 1) [91]. It is possible that SP and sphere formation are different progenitor phenotypes in different cell lines with different genetic makeups, including *VHL* and *TP53* mutant status (Table 2).

**Table 2:** Cell lines used in RCC stem/progenitor cell studies^a^

Cell line^b^	VHL status	Other significant tumorigenic genes mutated	Tumor type	Characteristics
769-P	mutant	ABC, BAP1, CXCL12, MAPK kinases, NCAM, PDGFR, VEGFC	Primary ccRCC	Tumorigenic in nude mice No lung metastases, tumors by SP
786-O	mutant	PTEN, TP53, FGFR, IL-1, MAPK kinases, VEGFC, ABC, FLT1	Primary ccRCC	NCI-60 panel^c^ Tumorigenic in nude mice; tumors by SP and lung metastases in xenografts
OS-RC-2	mutant	ABC, HLA, PBRM1, PTEN, WT1	Metastatic ccRCC	Tumorigenic in nude mice TP53 wild-type
Caki-1	wild-type	HIF1A, MAPK kinase, MET, MMP9, NCAM, VEGFC, ABC, FGFR, HLA, IL-10R, MAPK kinases, MET, NES, PIUK3, TRAF	Metastatic ccRCC	NCI-60 panel^a^ VHL and TP53 wild-type Sphere formation Tumorigenic, lung metastases Tumors with sarcomatoid changes by SP in xenografts
SN12C	wild-type	ABC, E-CDH, EGFR, PGF, TLR5, TP53, *KDM6A*	Mixed granular and clear cell morphology	NCI-60 panel^c^ VHL wild-type Tumorigenic in nude mice, liver metastasis
ACHN	wild-type	ABCA genes, MAPK kinases, TLR2, NCAM, MAPK kinases, PIK3, VEGFC	Metastatic mixed papillary and clear-cell RCC	NCI-60 panel^c^ VHL and TP53 wild-type Sphere formation Tumorigenic in nude mice Tumors by SP in xenografts
Caki-2	wild-type	ABC, EGF, FGFR, MAPK kinases, NCAM, PDGFR, VEGF, PBRM1	Primary cystic papillary	TP53 wild-type Tumorigenic in nude mice Tumors by SP
KRC/Y	wild-type	Mutated and overexpressed p53	Primary, cystic and necrotic, fibrous capsule, both clear and granular cells	VHL wild-type SP equally tumorigenic as Non-SP Sphere formation
RenCa	wild-type	n.d.^d^	Spontaneous renal cortical adenocarcinoma in BALB/c mice	VHL wild-type Tumorigenic in syngeneic mice
RCC-26	n.d.^d^	n.d.^d^	Primary stage-I ccRCC (T1, G2)	Non-tumorigenic in nude mice
RCC-53	n.d.^d^	n.d.^d^	Primary stage-IV ccRCC	Tumorigenic in nude mice
SMKT-R2	n.d.^d^	n.d.^d^	Primary mixed alveolar type and clear cell	Tumorigenic in nude mice
SMKT-R3	n.d.^d^	n.d.^d^	Primary papillary type and granular cell subtype	Tumorigenic in nude mice
SK-RC-39	n.d.^d^	n.d.^d^	Metastatic papillary RCC	Tumorigenic in nude mice
SK-RC-42	n.d.^d^	n.d.^d^	Metastatic RCC	Tumorigenic in nude mice

^a^Cell lines grouped based on the status of *VHL* gene (mutant or wild-type), and subgrouped according to the histology (ccRCC, light shade; non-ccRCC or mixed, darker shade)

^b^For pathological and genetic features of the cell lines, refer to Wolf et al. [92], Brodaczewska et al. [93], and Sanger Institute COSMIC database

^c^The NCI-60 cancer cell line panel is a group of ~ 60 human cancer cell lines used by the National Cancer Institute (NCI) for the screening of compounds to detect potential anticancer activity, which consists of most representative of in vitro model for the common cancer types

^d^n.d.: not determined

Therefore, attempts to identify CSCs from established malignant cell lines may be inherently problematic since these cells have accumulated numerous genetic modifications to adapt to in vitro monoculture conditions. In addition, these commonly used cell lines are derived from renal cancers of different histological features and genetic makeups. The characteristics of the various cell lines used in the above-summarized studies are listed in Table 2. It is quite often that different RCC cell lines are used without consideration for their pathological and genetic features [92, 93]. For example, ACHN is not a ccRCC cell line but a mixed papillary and clear-cell morphology, and does not harbor *VHL* loss-of-function mutations, and KRC/Y is of granular and clear-cell histology and also *VHL* wildtype. Their inclusion in the same study (Study 12 in Table 1) yielded opposite results, as discussed above. Caki-1 and Caki-2, although originally isolated from presumed ccRCC patients, are both *VHL*-positive, and Caki-2 cells and their derived tumors in fact exhibit characteristics of high-grade papillary RCC (pRCC) in their histology and gene expression patterns [94].

Therefore, the results from studies with use of only *VHL*^+^ ccRCC cell line (Studies 5 and 12 in Table 1) [72] or mixed use of *VHL*^+^ and *VHL*^*−*^ cell lines (Study 13 in Table 1) [91], or mixed use of ccRCC and pRCC cell lines (Studies 6, 13, and 14 in Table 1) [70, 90], are difficult to extrapolate with relevance to clinical ccRCC [95]. As such, clinical samples should offer a more realistic chance to identify genuine tumor progenitor cells.

Many studies using clinical samples as well as cell lines implicated CXCR4 as a marker for normal human renal progenitor cells and for the tumor progenitor cells in ccRCC (Studies 2, 3, 7, and 14 in Table 1) [68, 69, 71, 91]. The CXCR4^+^ subpopulation in patient-derived xeno-transplantable ccRCC cells display sphere-forming capacities and are more tumorigenic in comparison with their CXCR4^−^ counterpart. Notably, the expression of CXCR4 and its ligand, CXCL12/SDF-1α, is positively regulated by HIF that is stabilized and activated in *VHL*-deficient ccRCC cells [96, 97].

Canonically, CXCR4 is the receptor of the chemokine CXCL12/SDF-1 that induces metastasis [98]. HIF2α-induced expression of CXCR4 can also promote sphere formation and self-renewal of ccRCC cell lines [91]. In addition, CXCR4 can also enter the nucleus and interact with nuclear HIF-1α to enhance the expression of HIF target genes and promote ccRCC metastasis [99]. As such, elevated expression of CXCR4 is significantly associated with high-grade and advanced-stage ccRCC, as well as high rates of tumor recurrence [100]. Intriguingly, the significantly elevated CXCR4 mRNA levels were detected in primary ccRCC tumors without metastases, but not in metastasized tumor, and were correlated with short survival time [68]. This suggests that CXCR4 is a predictive marker for tumor aggression and metastasis, perhaps being involved in progenitor cell maintenance, but not contributing to metastasis directly. The notion is consistent with the finding that hypoxia is an important feature of stem/progenitor cell niche [101, 102].

It therefore appears that the best strategy for isolating TICs of ccRCC is to include known VHL-HIF targets in addition to stem cell markers from clinical samples, such as the studies of Addla et al. and Fendler et al. [71, 81], which both identified CXCR4, signaling pathways WNT (β-catenin) and NOTCH1, and stem cell marker CD133 and PAX2, as signature markers (Studies 3 and 8, Table 1).

### ccRCC initiation and progression

Sporadic ccRCC tends to be late onset [103, 104]. Modeling of ccRCC progression based on genomic data demonstrates that haploid chromosome 3p loss, likely in the renal stem/progenitor cells (RSPCs), occurs early in childhood or adolescence, representing an initiating genetic event that is followed by slow clonal expansion in the subsequent 5–20 years [41]. The RSPCs with initial loss of chromosome 3p can be regarded as pre-tumorigenic because although they may develop into tumor cells, these RSPCs with 3p loss are not fast-growing as proliferating tumor cells. Indeed, the initial expansion results in only a modest number of progenies (a few hundred cells). The TRAcking renal Cancer Evolution through therapy (Rx) (TRACERx) study suggests that inactivation of the second allele of *VHL* occurs after 3p loss and before subclonal evolution that leads to metastasis [41, 51]. Therefore, inactivation of the second allele of *VHL* likely marks the emergence of TICs and sets off tumor growth and subclonal evolution. There is a latency period of 10–30 years between the emergence of TICs and clinical diagnosis (Fig. 2). Hereditary ccRCC, as in the familial VHL disease patients, follows the similar genetic trajectory; but since these patients inherit the first *VHL* gene inactivation mutations in the germline, the clinical diagnosis of ccRCC is years to decades earlier. Based on this tumor initiation-coupled subclonal evolution model (Fig. 1), one can envisage the difference between tumors with low primary heterogeneity and rapid malignant progression, and those with high primary heterogeneity and attenuated malignant progression (Fig. 2). This model also provides a reasonable explanation for the difference between TICs and MICs; that is, the initial 3p loss combined with loss of the second *VHL* allele can be viewed as the cause of TIC emergence. Following the appearance of TICs, metastatic subclones can emerge via different genetic and/or epigenetic events. Therefore, if the starting materials for isolating CSCs are malignant tumor mass or established malignant cell lines, it is likely that different “CSC” markers will be identified, reflecting the diverse genetic makeups of different metastatic subclones. On the other hand, TICs can offer a more homogeneous marker set for early diagnosis and treatment targets.

These findings also suggest that the TICs of ccRCC may indeed be the mutated adult RSPCs, since ccRCC appears to originate from a very limited cell population that expands to only a few hundred cells when second hit on the *VHL* allele occurs. The existence of RSPCs has been suspected since adult kidney is under constant chemical and mechanical assaults, and tubule repair is a well-controlled process [105–108]. Acute tubular injury can result in extensive tubule epithelial cell death, which is usually followed by a regenerative response characterized by epithelial cell proliferation [109, 110]. Such repair and regeneration processes involve the activation of stem/progenitor cells.

RSPCs are difficult to identify because of the complexity of the kidney structures and the complex developmental process. There are up to 26 cell types in mammalian adult kidney according to one study [111], including 16 different specialized epithelial cell types [112]. Some recent single-cell analyses have even identified 41 cell populations of renal lineage and 32 of non-renal lineage in the adult kidney [113], although whether these renal lineages are all functionally distinct is not clear.

During embryonic development, the nephrons are constructed from existing epithelia (from ureteric buds to form collecting ducts) and from metanephric mesenchyme via the process of mesenchymal-to-epithelial transition (to form distal and proximal tubules, and Bowman’s capsules) [111, 114, 115]. It has been suggested that each distinct segment of the renal tubule system can possess its own adult progenitor cells. Alternatively but not exclusively, a special group of progenitor cells can repopulate other, more distant regions of the nephron via migration, proliferation, and differentiation. Indeed, different adult renal progenitor cells have been identified [116, 117]. A few studies have also identified potential kidney progenitor cells in the interstitial tissue or mesenchyme [118, 119]. These studies are summarized in Table 3. Mostly, these studies employed functional assays such as label (BrdU)-retention (Studies 1 and 2 in Table 3) [120, 121], limiting dilution for proliferative capacity (Studies 3 and 4 in Table 3) [122, 123], serial dilution for in vitro culture (Study 5 in Table 3) [124], outgrowth of cultured kidney tissues (Studies 6–8 in Table 3) [74, 75], and SP isolation (Study 9 in Table 3) [125]. Some other studies used preconceived stem cell markers for isolation (Studies 10 and 11 in Table 3) [73, 126, 127]. More recently, single-cell RNA sequencing (scRNAseq) was used to identify kidney stem cells from urine (Study 12 in Table 3) [128]. These studies largely confirmed the presence of known stem cell markers such as Yamanaka factors, CD133, CD44, CD24, CD106, Sca-1, etc. in the presumptive kidney stem/progenitor cells, which supports using some of these markers for identifying or validating the presence of TICs in RCC.

**Table 3:** Prior studies on kidney stem/progenitor cells

Studies	Approaches	Markers	Other markers	Negative expression	Location/cell type	Property and function	Other characteristics	Reference
1	Label (BrdU)-retaining cells in rat kidney				PT and DT; thick ascending LOH; CD	Proliferation after injury in vivo		Maeshima et al. [120]
2	Label (BrdU)-retaining cells in rat and mouse kidneys				Renal papilla	Multipotency in vitro; sphere formation; proliferation after injury; incorporate into parenchyma after injection into renal cortex	Scattered LRCs also found in outer cortex and medulla	Oliver et al. [121]
3	Limiting dilution for proliferative mouse kidney cells	Sox9 (used for lineage tracing)	CD133, Lgr4, Foxd1, Pax8, Notch (Hey1, Hes1, Hes5) and Wnt (Axin) target genes	Lgr5, Pax2, Six2, Scf, c-Kit, CD90, and CD105	PT and DT	Populate PT, LOH, and DT in embryo and after injury in adult (but not glomeruli or CD)		Kang et al. [122]
4	Limiting dilution and growth of dissected rat kidney S3 segment		Sca1, c-Kit, c-Met, Vimentin, Wnt4, WT-1 Pax2		S3 segment of the proximal tubule in rat kidney	Form tubule structure after implantation; regenerate tubules after drug-induced injury	Differentiate into tubule cells expressing multiple segmental markers	Kitamura et al. [123]
5	Serial passages of cultured whole adult rat kidney suspension	Oct4 (used for lineage tracing)	Pax2, CD90, CD44, Vimentin	SSEA-1, CD133, CD106, CD31	PT in the cortical medullary junction	Multipotency in vitro; incorporate into PT and DT (but not LOH) after injection into injured kidney		Gupta et al. [124]
6	Outgrowth of cultured capsulated glomeruli in vitro followed by sorting	CD133^+^ CD24^+^, CD106^+^ (used for sorting)	CK7 and Vimentin		Urinary pole of Bowman's capsule	Proliferate and differentiate into podocyte and tubular lineages in vitro; regenerate podocytes and tubular cells in SCID post-injuries		Angelotti et al. [74]
7	Outgrowth of cultured renal tubules in vitro followed by sorting	CD133^+^, CD24^+^, CD106^−^ (used for sorting)	CK7 and Vimentin		PT, DT	Proliferate and differentiate into tubular lineage in vitro; regenerate tubular cells in SCID post-injuries		Angelotti et al. [74]
8	Outgrowth of cultured human glomeruli in vitro followed by sorting	CD133, CD24 (used for sorting)	CD106, CD105, CD54, and CD44		Urinary pole of Bowman's capsule	Self-renewal; clonogenic potential; multilineage differentiation; repopulate glomeruli, PT, and DT in SCID mice after injury	• Differentiate into tubule cells expressing multiple segmental markers simultaneously in vitro	Sagrinati et al. [75]
9	Side population of adult mouse whole kidney cells after Hoechst 33342 staining	CD24a (used for localizing SP cells in vivo)	Sca1, CD105, CD44, Pax8, Notch1/2	CD45, CD34, and c-Kit (positive for SP from bone marrow)	Mostly in PT, also in DT, thick ascending LOH, CD	Multipotency in vitro; incorporate into explant of developing MM and UB; engraft into PT, DT, and CD of injured adult kidney		Challen et al. [125]
10	Magnetic bead sorting of human adult renal papilla suspension, followed by colony formation in culture	CD133	OCT4, CD73, CD29, CD44, CD146, SSEA-4, ZO-1, cytokeratin, Vimentin, Nestin, PAX2 OCT4, c-MYC, KLF4	CD34, CD90, CD117, CD45	Papilla, LOH thin limb segments	Differentiate into tubule cell fates of all segments in vitro; incorporate into different tubule segments after injection into SCID mice		Bussolati et al. [73] Bussolati et al. [184]
11	MSCs isolated from adult mouse kidney; sorting for Lin^−^CD31^−^ CD24^lo^ Sca-1^+^; sorting for Hob7-driven GFP^+^ cells; lineage tracing	CD24^lo^ Sca-1^+^	Hoxb7, Wnt4, BNP, Uroplakin 1b, Aqp2 (principal cell marker)	F4/80 (macrophage marker), UMOD; CD31; Pendrin or AE1 (intercalated cell markers); PDGFRα and β (pericyte markers)	CD (principal cells)	Display EMT after in vitro culture; exhibit MSC-like property; integrate into Aqp 2-positive medullary CD when injected into neonatal kidneys; form epithelial structures in vitro and in vivo	May originate from interstitial Wnt4*-*expressing cells integrated into CD after birth; able to produce growth factors that promote epithelial wound repair in vitro	Li et al. [127]
12	scRNAseq of human urine cells	SOX9 (used as marker in selection for serial enrichment)	SOX4, HES1, TLE4		Un-specified tubular cells	Serial culture enrichment for SOX9^+^; incorporate into incised SCID mouse kidney		Wang et al. 2021 [128]

*CD* collecting duct, *DT* distal tubule, *LOH* loop of Henle, *LRC* label-retaining cell, *MSC* mesenchymal stem cell, MM metanephric mesenchyme, *PT* proximal tubule, *UB* uretic bud

Therefore, these studies, in aggregate, indicate that RSPCs exist in different segments of the renal tubule systems. They all exhibit canonical stem/progenitor cell activity such as multipotency and clonogenic activity in prolonged culture in vitro, and can repopulate tubule epithelia in kidney injury models. Although they all express a common set of stem/progenitor markers, each subpopulation may exhibit differences in specific marker gene expression. This may explain the clinical and experimental observations that although proximal tubule cells are the main origin of ccRCC, other renal tubule origins such as distal tubule and subregions of collecting duct can also give rise to ccRCC [40, 129]. Many of these RSPCs express NOTCH and/or WNT signaling signatures (Studies 3, 4, 9, 11, and 12 in Table 3). Interestingly, NOTCH and WNT signaling pathways appear to be important factors for specifying cells with tumor-initiating capacity identified from clinical cohorts that mainly include early-stage ccRCC samples [71, 81] (Studies 3 and 8 in Table 1). As such, a rational approach to validate the TICs of ccRCC may be to inactivate *VHL* specifically in one of these RSPCs, and examine the tumor-initiating property of the resultant mutant progenitor cells.

### Loss of *VHL* and ccRCC initiation

In sporadic ccRCC, the first genetic event is often the haploid 3p loss that generates heterozygous loss of *VHL, SETD2, PBRM1*, and *BAP1*. The TICs then emerge after the loss of the second *VHL* allele, usually as a result of deletion, loss-of-function mutation, or epigenetic inactivation of gene expression. In the hereditary form of ccRCC that occurs in the familial *VHL* disease, the genetic/epigenetic events are reversed. That is, the patients first inherit *VHL* inactivating genomic mutations, then acquire loss-of-heterozygosity via 3p loss or epigenetic alterations. Therefore, biallelic loss of *VHL* appears to be the essential requirement for ccRCC initiation, the rare wild-type *VHL* ccRCC notwithstanding, and the 3p loss can occur before or coincidental with the second *VHL* allelic loss. Haploid 3p loss likely serves as an auxiliary oncogenic change that facilitates the subclonal evolution. Indeed, although haploid 3p loss is found in 90% of ccRCC cases, biallelic losses of *PBRM1*, *SETD2*, and *BAP1* are only found in ~ 30–40%, 11%, and 10% of ccRCC cases, respectively [8, 12]. It is possible that haploid-insufficiency of *PBRM1*, *SETD2*, and *BAP1* resulting from 3p loss can lead to epigenetic changes and facilitate acquisition of the additional hits that lead to malignancy. Indeed, heterozygous 3p loss is not unique to ccRCC; it is found in a significant number of cases in head and neck, breast, and ovarian cancers [130–132]. We suggest that the RSPC with biallelic *VHL* loss can be considered TIC of ccRCC. The question then is how loss of *VHL* function can set off the pathogenic process that leads to growth of ccRCC?

*VHL* is not a typical tumor suppressor gene such as *TP53*, *PTEN,* or *Rb* that directly regulates cell death or proliferation. However, based on previous studies, by acting as a scaffold protein, pVHL does indirectly regulate several key events related to tumor progression. These oncogenic events, when occurring in RSPCs, can induce the formation of TICs.

1. Proliferation. One of the earliest findings concerning the function of *VHL* is that TGF-α is upregulated in *VHL* mutant cells [133], which can lead to autocrine activation of the PI3K and ERK signaling pathways, two canonical inducers of cell proliferation. Also important, pVHL can suppress regulatory-associated protein of mTOR (RAPTOR) thus reducing the mTOR signaling [134]. Since mTOR is a major inducer of cell growth and proliferation, loss of *VHL* function can lead to increased mTOR signaling and tumor growth [6, 8]. Furthermore, the most salient characteristic of the *VHL* mutant cells is the hypoxic response induced by the stabilization of HIF-α, which results in tumor angiogenesis [via overexpression of vascular endothelial growth factor (VEGF) and Oncostatin M (OSM)] and metabolic switch (via reduced oxidative phosphorylation-based respiration) [23, 135, 136]. Both of these changes are critical for tumor growth. Furthermore, loss of pVHL can suppress cyclin-dependent kinase inhibitor p27kip1 that is involved in cell-cycle arrest [137].
2. Apoptosis. It has been documented that *VHL* can inhibit apoptosis via Bcl-2 signaling, suggesting that *VHL* inactivation can lead to increased cell death [138]. Conversely, other studies indicate that *VHL* deficiency can promote survival and proliferation via activation of HIF-1α and other factors [139, 140]. Such discrepancy may be related to the differential functions of HIF-α isoforms [141, 142]. On the other hand, pVHL can promote apoptosis in a HIF-independent manner by stabilizing p53 via suppressing Mdm2-mediated ubiquitination and nuclear export of p53. In addition, pVHL can increase p53 acetylation, and hence activity, by p300 under genotoxic stress [143]. The net result is the destabilization and decreased activity of p53 in *VHL*-deficient cells. Therefore it is possible that under stress conditions, *VHL* loss-of-function does confer cell survival advantages.
3. Genome instability. Genome instability is a distinguishing feature of tumor cells [144], which is important for acquiring necessary mutations that promote the formation of metastatic subclones. ccRCC is not an exception, but its mutational burden is less severe compared with other cancers [145]. Indeed, ccRCC cells do not contain mutations in DNA damage response genes such as *BRCA1/2* or mismatch repair genes. These observations indicate that ccRCC may possess a unique mechanism for generating genome instability. One possible mechanism is related to the microtubule-stabilizing activity of pVHL [146, 147]. Thus, loss of *VHL* function can result in spindle malformation during cytokinesis, leading to chromosome instability [148]. In addition, pVHL can induce DNA damage repair of double-stranded breaks via generation of K63-linked polyubiquitin chains [149] that bind to damaged DNA and recruit repair enzymes [150]. Loss of *VHL* results in less efficient repair of DNA double-stranded breaks. Interestingly, it has also been reported that loss of *PBRM1*, another frequently mutated gene in ccRCC, can relieve the severe stress of DNA damages caused by *VHL* loss [151], thus providing a mechanistic explanation for the frequent coexistence of *VHL* and *PBRM1* losses.
4. Reconstitution of microenvironment. It has been shown that tumor microenvironment plays a critical role in promoting tumor growth and immune evasion [152–154]. In particular, many forms of cancer, including ccRCC, have been linked to chronic tissue inflammation [21, 155–157]. It has recently been demonstrated that loss of *VHL* can generate a hypoxic niche for tumor progenitor cell maintenance [102]. Our laboratory has shown that loss of *VHL* function can also induce inflammatory response via intracellular ER stress [21]. The inflammatory response results in secretion of TNFα family of cytokines including IL-6 and OSM that induce alternatively activated macrophages and inflammation of vascular endothelia, respectively [22, 23]. The activated macrophages and endothelial cells in turn induce immune suppression and tumor cell EMT via the expression of PD-L1 and chemokines such as CCL18. The above notions are further explored in the following section.

### Effects of microenvironment

Stem cells are known to require specialized niches for maintenance. CSCs have also been proposed to reside in a specialized niche, consisting of stromal cells such as cancer/carcinoma-associated fibroblasts (CAFs), endothelial cells, immune modulating cells including macrophages and myeloid-derived suppressor cells, reconstituted extracellular matrix, and cytokine-containing extracellular vesicles [158–160].

In this sense, *VHL* deficiency may be a unique self-fulfilling cellular characteristic for generating niches suitable for stem/progenitor cells, since loss of *VHL* function leads to HIF stabilization, resulting in hypoxic responses that can induce angiogenesis and reconstitute the microenvironment [102, 161]. It has also been known that ccRCC progression is strongly associated with chronic inflammation [162]. Such inflammatory microenvironment can facilitate the growth and malignant transformation of tumor cells [22, 23]. In particular, results from our and other laboratories have shown that hypoxic environment containing *VHL*-deficient kidney cells can attract monocytes and induce macrophage differentiation via overproduced IL-6, TGF-β, and VEGF [22, 154, 163], which in turn coordinate maintenance and activation of CSCs/TICs [160, 164]. *VHL* mutant cells also activate endothelial cells that favor inflammatory reactions via overproduced VEGF and OSM [23], which may also serve as the vascular niche that is a widely-recognized component of stem cells and CSC niche [165]. *VHL* mutant cells also overproduce PDGF-B that activates CAFs in a HIF-independent and Sp1-dependent manner [166]. CAFs produce VEGF, PDGF, TGF-β, EGF, FGF, HGF, CXCL12/SDF-1, and osteopontin that promote EMT and induce angiogenesis important for CSC maintenance. Other less well-studied potential CSC niche components such as mesenchymal stem cells, neurons, lymphatics, etc., require further elucidation.

Besides the cellular components, CXCR4 and CXCL12/SDF-1 expression is also induced by hypoxia in TICs or stromal cells [96, 97], potentially facilitating the mobilization of stem cells. Furthermore, *VHL* mutant cells are known to overproduce fibronectin and collagens [167–170] that enrich the extracellular matrix (ECM), lysyl oxidase that crosslinks the collagen fibers [171, 172], and metalloproteases (MMPs; mainly MMP2, MMP9, and MT1-MMP) that remodel the ECM [173–175]. Therefore, although it is not yet known whether RSPCs reside in specialized niches, it is entirely possible that the importance of *VHL* inactivation in initiating ccRCC is that it can create a favorable microenvironment for the emergence of TICs.

As such, crosstalk between TICs of ccRCC and the components of the microenvironment is a critical aspect of TIC development and maintenance [176]. Such interaction is usually mediated through cytokines or growth factors, but recently, metabolites such as methionine have also been shown to promote CSC/TIC maintenance in a paracrine manner [177, 178]. Interestingly, in ccRCC, methionine can be supplied by a subpopulation of pericytes expressing platelet-derived growth factor receptor-beta (PDGFR-β) and G-protein-coupled receptor 91 (GPR91), which are activated by succinate secreted by the TICs and received by GPR91 on pericytes [179].

In the case of ccRCC metastasis, in addition to the contributing stromal components described above, it has been shown that 9p21.3 loss is a common event in metastatic subclones [41]. 9p21.3 encompasses tumor suppressor genes *CDKN2A/B* and the *Type I Interferon* (*IFN*) gene cluster. Interestingly, 9p21.3 loss has been found in 14 different malignant cancer types based on analysis of The Cancer Genome Atlas data [180]. In a syngeneic mouse model of pancreatic cancer, functional genetic study indicates that while loss of the *CDKN2A/B* genes is important for tumor growth, deletion of the *Type 1 IFN* locus is specifically needed for metastasis [181]. However, if these cancer cells were injected directly into circulation, deletion of the *Type 1 IFN* cluster no longer offered advantages in metastasis over the *Type 1 IFN*-positive counterparts [181]. This suggests that the consequence of *Type 1 IFN* loss is alteration of the immunogenic response in the microenvironment, thus effecting malignant tumor progression. As such, MICs of ccRCC may be suppressed by reactivating the immune cells induced by Type 1 IFN.

## Conclusions and perspectives

**Fig. 3:**
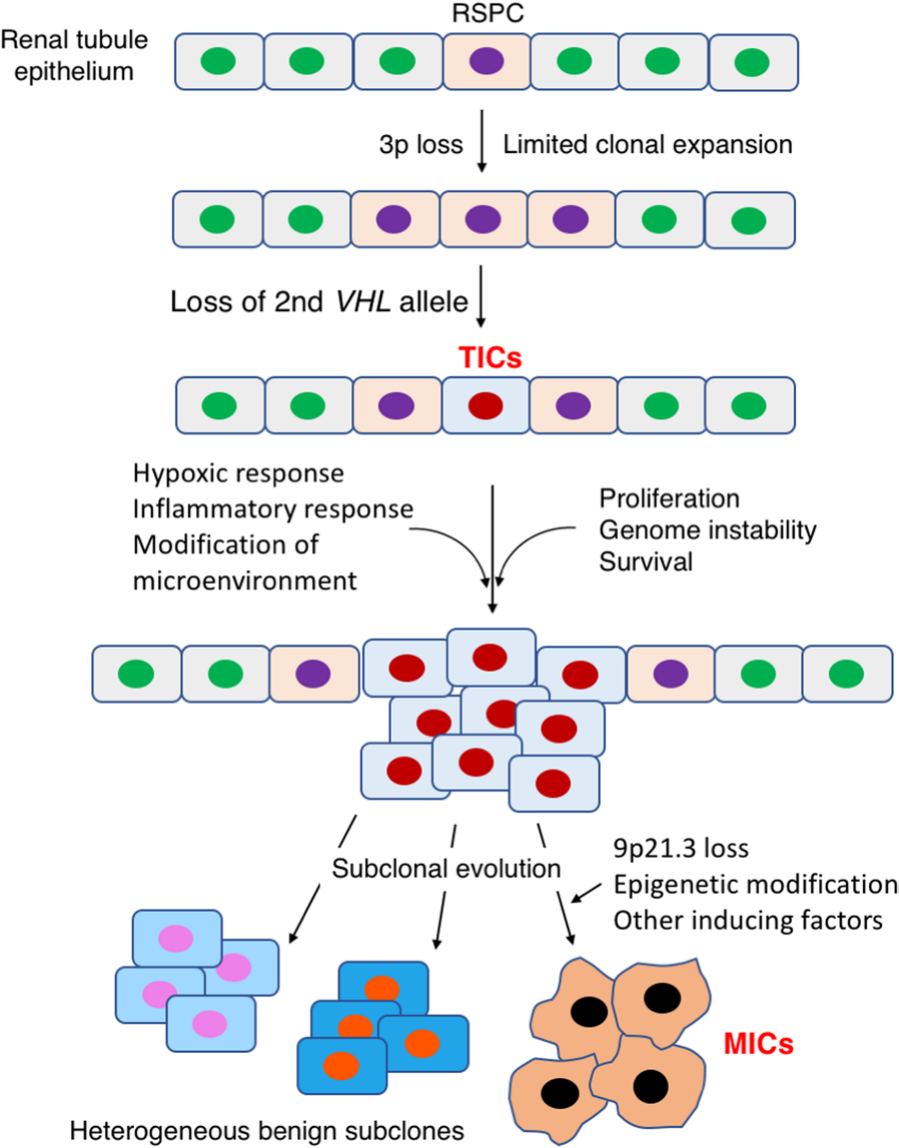
Model of tumor initiation and metastasis initiation of ccRCC. Renal stem/progenitor cell (RSPC) experiences chromosome 3p loss and begins a slow clonal expansion, followed by loss of the 2nd *VHL* allele, and becomes tumor-initiating cells (TICs). Loss of *VHL* function promotes proliferation, survival, genome instability, and reconstitution of the microenvironment, resulting in subclonal evolution, which mainly produces heterogeneous subclones of benign tumor cells. The subclonal evolution may also be aided by epigenetic changes enhanced by the loss of haploid *PBRM1*, *SETD2*, and *BAP1*. Chromosome 9p21.3 loss and other genetic events such as EMT induction then generate metastasis-initiating cells (MICs)

In summary, ccRCC initiation is unique in that it requires, at a minimum, only loss of *VHL* function. This is achievable because pVHL as a scaffold protein can participate in multiple cellular functions involved in different aspects of tumorigenesis [182]. Figure 3 shows the model that explains the origin of TICs of ccRCC and the formation of MIC subclone. Loss of *VHL* in normal kidney tissue progenitor cells confers TICs the tumor-initiating capacity. These cells can be recognized by the markers of tissue progenitor cells such as NOTCH or WNT signaling components, and progenitor cell marker CD133, PAX2, or CD105, in addition to the VHL-HIF signaling target CXCR4, and urine/serum inflammatory markers such as KIM-1. MICs then emerge after intrinsic genetic changes such as 9p21.3 loss and/or epigenetic changes promoted by haploid loss of *PBRM1*, *SETD2*, and *BAP1.* Extrinsic factors such as cytokines, growth factors, and metabolites emanated from the microenvironment can further induce metastatic transformation.

The question remains as to why this unique genetic condition only occurs in ccRCC and a few other cases of benign tumors, but not in other cancers. There are two possibilities. First, the combination of genetic and physiological conditions required for ccRCC formation is only suited for the kidney microenvironment, and even only in certain populations of the kidney epithelial cells [40]. The unique kidney microenvironment may include the unique set of resident macrophages that can be induced by *VHL* mutant cells [183]. Alternatively but not exclusively, *VHL* mutant cells may not survive (and therefore no tumor growth) in other tissues. One scenario may be that cells with genome instability resulting from *VHL* loss can survive better in kidney because these cells have a robust DNA damage response program a priori. Thus the balanced cell survival and accumulation of mutations may be the key to ccRCC development. More detailed analyses of the cellular and molecular characteristics of the TICs of ccRCC should answer this question. Understanding the origin of the TICs and MICs for ccRCC should offer a novel avenue for early detection and prevention of malignant transformation of this deadly disease.

## Data Availability

All data analyzed in this review have been published in primary research articles cited as references.

## Abbreviations

ABC: ATP-Binding Cassette
ALDH1A1: Aldehyde Dehydrogenase 1 Family Member A1
APC: Adenomatous polyposis coli
AQP2: Aquaporin 2
BAP1: Brca1-Associated Protein 1
BMI: B-Cell Lymphoma Murine Leukemia Virus Integration Site
BNP: B-Type Natriuretic Peptide
BrdU: Bromodeoxyuridine/5-bromo-2'-deoxyuridine
CAF: Cancer/Carcinoma-Associated Fibroblast
c-MYC: Cellular myelocytomatosis
ccRCC: Clear-cell renal cell carcinoma
CD: Cluster of differentiation
CK: Cytokeratin
CKD: Chronic kidney disease
CSC: Cancer stem cell
CXCR4: C-X-C Chemokine Receptor Type 4
DLL1/4: Delta-like protein 1/4
ECM: Extracellular matrix
EGF: Epithelial growth factor
EMT: Epithelial-to-mesenchymal transition
FACS: Fluorescence-activated cell sorting
FGF: Fibroblast growth factor
FOXD1: Forkhead Box D1
FU: Fluorouracil
JAG1/2: Jagged canonical notch ligand 1/2
GATA4: GATA binding protein 4
GPR91: G Protein-Coupled Receptor 91
Gy: Gray unit of ionizing radiation
HES1: Hairy and enhancer of split-1
HEY1: Hairy Ears, Y-Linked 1
HGF: Hepatocyte growth factor
HIF: Hypoxia-inducible factor
IFN: Interferon
IL-6: Interleukin 6
KIM-1: Kidney injury molecule-1
KLF4: Krüppel-like factor 4
LRC: Label-retaining cell
LGR5: Leucine Rich Repeat Containing G Protein-Coupled Receptor 5
MDR1: Multidrug resistance protein 1
MHC-II: Major histocompatibility complex class II
MIC: Metastasis-initiating cell
MMC: Mitomycin C
MMPs: Matrix metallopeptidases
MSC: Mesenchymal stem cell
MTX: Methotrexate
MYST3: Histone Acetyltransferase (Monocytic Leukemia) 3
NANOG: Homeobox Transcription Factor Nanog
NGAL: Neutrophil Gelatinase-Associated Lipocalin
NOD/SCID: Non-obese Diabetic/Severe Combined Immunodeficiency
NOTCH1/2: Neurogenic Locus Notch Homolog Protein 1/2
OCT3/4: Octamer-Binding Transcription Factor 3/4
OSM: Oncostatin M
PAX2/8: Paired Box 2/8
PBRM1: Polybromo 1
PDGF: Platelet Derived Growth Factor
PDGFR: Platelet Derived Growth Factor Receptor
PROM1: Prominin 1
RCC: Renal Cell Carcinoma
RSPC: Renal Stem/ Progenitor Cell
RTK: Receptor Tyrosine Kinase
SALL1: Spalt Like Transcription Factor 1
SCA1: Stem Cells Antigen-1
SCF: Stem Cell Factor
scRNAseq: Single-Cell RNA Sequencing
SDF-1: Stromal-Derived Factor-1
SETD2: SET Domain-Containing 2
SIX2: Sine Oculis Homeobox Homolog 2
SNAI1: Snail Family Transcriptional Repressor 1
SOX2/4/9: SRY-Box Transcription Factor 2/4/9
SP: Side Population
SSEA-4: Stage-Specific Embryonic Antigen 4
TGF-α: Transforming Growth Factor-alpha
TGF-β: Transforming Growth Factor-beta
TIC: Tumor-Initiating Cell
TWIST1: Twist Family bHLH Transcription Factor 1
TLE4: Transducin-Like Enhancer of Split 4
UMOD: Uromodulin
VCAM1: Vascular Cell Adhesion Molecule 1
VEGF: Vascular Endothelial Growth Factor
VEGFR2: Vascular Endothelial Growth Factor Receptor 2
VHL: Von Hippel–Lindau
WNT: Wingless-Related Integration Site
WT-1: Wilms Tumor 1
ZEB1/2: Zinc Finger E-Box Binding Homeobox 1/2
ZO-1: Zonula Occludens 1
